# Immunomodulation by Mesenchymal Stromal Cells and Their Clinical Applications

**DOI:** 10.15436/2471-0598.17.022

**Published:** 2017-04-10

**Authors:** Joaquin Cagliani, Daniel Grande, Ernesto P Molmenti, Edmund J. Miller, Horacio L.R. Rilo

**Affiliations:** 1 The Feinstein Institute for Medical Research, Center for Heart and Lungs, Northwell Health System, Manhasset, N Y, USA; 2 The Elmezzi Graduate School of Molecular Medicine, Northwell Health System, Manhasset, NY, USA; 3 The Feinstein Institute for Medical Research, Orthopedic Research Laboratory, Northwell Health System, Manhasset, N Y, USA; 4 Transplantation of Surgery, Department of Surgery, Northwell Health System, Manhasset, NY, USA; 5 Pancreas Disease Center, Department of Surgery, Northwell Health System, Manhasset, NY, USA

**Keywords:** Mesenchymal stromal cells, Immunomodulation, Immunosuppression, Cell therapy, Clinical trials, Animal studies

## Abstract

Mesenchymal stromal cells (MSCs) are multipotent progenitor cells that can be isolated and expanded from various sources. MSCs modulate the function of immune cells, including T and B lymphocytes, dendritic cells, and natural killer cells. An understanding of the interaction between MSCs and the inflammatory microenvironment will provide critical information in revealing the precise *in vivo* mechanisms involved in MSCs-mediated therapeutic effects, and for designing more practical protocols for the clinical use of these cells. In this review we describe the current knowledge of the unique biological properties of MSCs, the immunosuppressive effects on immune-competent cells and the paracrine role of soluble factors. A summary of the participation of MSCs in preclinical and clinical studies in treating autoimmune diseases and other diseases is described. We also discuss the current challenges of their use and their potential roles in cell therapies.

## Introduction

Mesenchymal stromal cells (MSC) were initially discovered by Friedenstein et al. in the mid-1970s as the small fraction of heterogeneous cells from the bone marrow that are readily isolated^[1]^. The cells were described as spindle-shaped cells which adhere to tissue culture surfaces and rapidly expand in culture.

During the 1980s the multilineage potential of MSCs were described by Piersma et al^[2,3]^ and Pittenger et al.^[4]^ based on their ability to differentiate into distinct mesenchymal cell lineages, including chondrogenic, adipogenic, osteogenic and even myoblast. However, these cells do not meet the specified stem cell criteria such as *in-vivo* demonstrations of long-term survival with self-renewal capacity^[5]^. Therefore, the International Society for Cellular Therapy (ISCT) had stated that these fibroblast-like plastic-adherent cells, regardless of the tissue of origin, should be termed “multipotent mesenchymal stromal cells” and retain the acronym “MSCs”^[6]^. Since then, the Mesenchymal and Tissue Stem Cell Committee of the International Society of Cellular Therapy proposed a minimum set of criteria to define MSCs. First, MSCs must be plastic-adherent during culture and present a fibroblast-like shape. Second, MSCs must present a specific immune phenotype by the expression of surface molecules CD105, CD73 and CD90, and not CD45, CD34, CD14 (or CD11b), CD79 alpha (or CD19) or human leukocyte antigen (HLA)-DR molecules. Finally, MSCs must have the *in vitro* capacity for trilineage mesenchymal differentiation. Thus, have the potential to differentiate *in vitro* into osteoblasts, adipocytes and chondroblasts^[7]^.

Although initially isolated from the bone marrow, MSCs were subsequently obtained from multiple adult and fetal sources, including the skin, muscle, kidney, dental pulp, spleen and heart. However, adipose tissue and the umbilical cord, represent major alternative sources to bone marrow due to the easy accessibility with minimal invasive methods^[8,9]^.

In recent years, several studies have extensively investigated the immunosuppressive potential *in vitro* and *in vivo* of MSCs^[10]^. These cells are an extraordinary model for investigating the biological mechanisms that allow a cellular population to generate diverse cell type. Furthermore, they are potential tools in cellular therapies for several clinical applications, such as those in which the immune response is exacerbated, diabetes^[11]^ and graft-versus-host-disease^[12]^.

Considering the significant advances reported in the field, this review addresses the current knowledge of the biological aspects involved in MSC immune regulatory capacity and the clinical focus of these characteristics in the treatment of several diseases with an immune component involved. We also summarize the preclinical and clinical studies of MSCs and emphasize the current knowledge on diseases for which MSC’s are a key component of cell therapy procedures. This review culminates with the current limitations in our understanding that may be the impetus for future studies.

## MSC’s and the Innate and Adaptive Immune System

**Figure 1:**
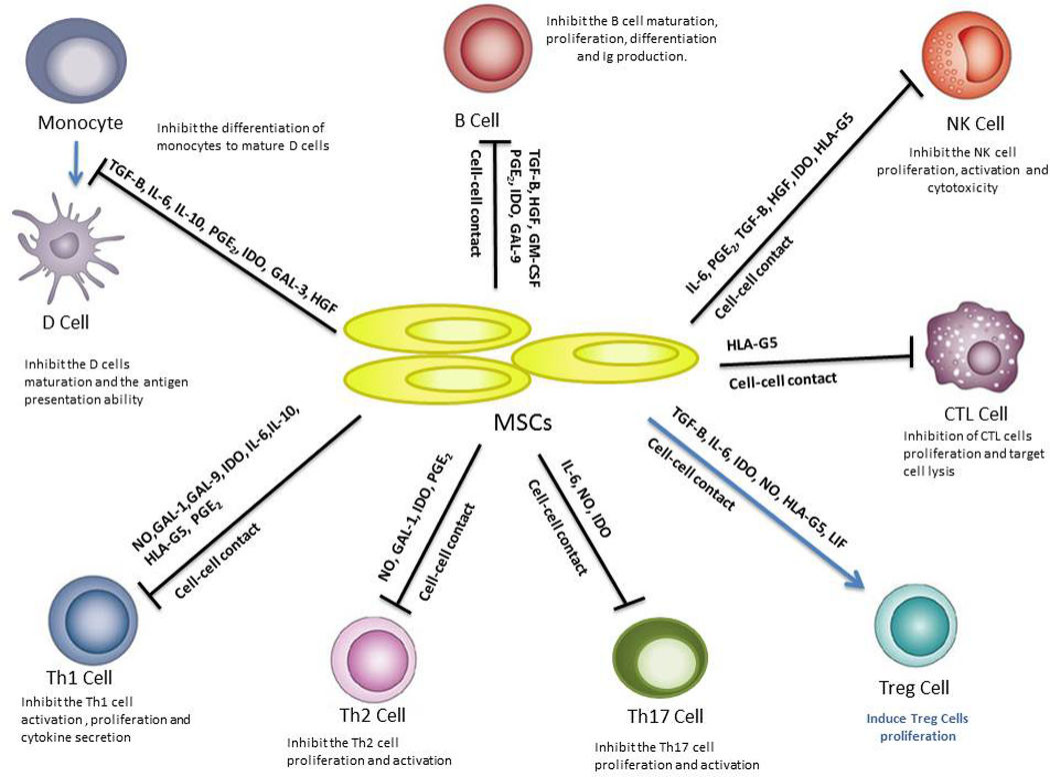
Immumodulatory effects of mesenchymal stem cells (MSC) on immune cells MSCs inhibit the monocyte differentiation into dendritic cells (DCs), suppress the activation and proliferation from B and Th1, Th2 and Th17 cells, induce the activity of T regulatory (Treg) and inhibit the proliferation and cytotoxicity of natural killer(NK) cells and cytotoxic T lymphocytes (CTL) cells through cell-cell contact mechanisms and through soluble factors.

Although the underlying mechanisms of MSC immunomodulation have yet to be elucidated^[13]^, they are likely mediated by the secretion of soluble factors and cell contact-dependent mechanisms in response to immune cells (Figure 1). Several studies have shown that MSCs regulate the adaptive and innate immune systems by suppression of T cells, generation of regulatory T cells, reducing B-cell activation and proliferation, maturation of dendritic cells, and inhibiting proliferation and cytotoxicity of NK cells^[14]^. Below, we describe and illustrate the immune regulatory effects of MSCs on specific immune cells (Figure 1).

## Cell to Cell Immunosuppressive Effects

### MSCs and T Lymphocytes

T lymphocytes play a central role as the major executor of the adaptive immune system response. Their functional properties are central to antigen specificity and memory associated with cognate immunity. In several studies MSCs have been shown to have potent anti-inflammatory and immune-modulating properties over T-cell activation, proliferation, differentiation and effector function^[15,16]^. This immunomodulation may be direct or may occur indirectly via modulatory effects on antigen-presenting cells such as dendritic cells (DCs), resulting in altered cytokine expression and impaired antigen presentation^[17–19]^.

During the activation of T lymphocytes, several studies have observed that bone marrow derived MSCs (BM-MSCs) prevent the expression of the early activation markers CD25 and CD69 in T cells stimulated with phytohemagglutinin (PHA)2^[20,21]^, whereas other studies describe no effect by BM-MSCs on the expression of these molecules^[22,23]^. Duffy MM et al proposed that such contradictory results may result from differences in the population of T cells studied^[24]^.

The immunosuppressive effects of MSCs on the proliferation of T cells has been confirmed by *in vitro* and *in vivo* studies^[25,26]^. It has also been shown that it is independent of the activation method^[27]^. However, the direct contact between MSCs and T lymphocyte necessary for the inhibition of T-cell proliferation remains controversial. Some authors have suggested that MSCs act via an immunosuppressive mechanism independent of cell to cell contact^[28]^. Whereas others have indicated that contact is required for efficient immune regulation^[29]^. Gao et al have recently proposed that in order to provide a pleiotropic immunomodulation that is responsive to different stimulants and that targets different immune cells, MSCs are likely to employ both direct cell to cell contact and soluble factors that complements for diverse and strong immunomodulation^[30]^.

Once the T cells are activated they can differentiate into the well described subsets T helper type 1 cells (Th1), T helper type 2 cells (Th2),T helper type 17 cells (Th17), T regulatory cells (Treg) and cytotoxic T lymphocytes (CTL) to perform their function. The differentiation of Th cells into effector cells depends largely on the cytokine milieu present at the time of antigen presentation and activation. Several studies have suggested that MSCs modulate the differentiation, function and balance of these subpopulations and foster the development of anti-inflammatory immune response.

### T helper type 1 cells (Th1)

In the presence of IL-1B, IL-27 and interferon-gamma (IFN-y) CD4+ T cells are differentiated into Th1 cells^[24]^. Once differentiated, Th1 cells activates and recruit macrophages to sites of inflammation through the release of IFN-y and TNF^[15,31,32]^. Th1 also induces immunoglobulin (Ig) G2 production by B cells^[33]^. Examples of dysregulation of Th1 are Type 1 Diabetes, systemic lupus erythematosus (SLE) and inflammatory bowel syndrome^[34,35]^.

When in contact with MSCs, several *in-vitro* and *in-vivo* studies have indicated that MSC presents primarily suppressive effects on Th1 cells differentiation and effector function^[36–38]^. Madec et al. demonstrated that MSC’s protect non-obese diabetic mice from diabetes by induction of IL-10 producing FOXP3+ Treg^[39]^. In a rat model, Boumaza et al showed that MSCs were associated with increased IL-10 and IL-13 expression by Tcells with increased frequencies of both CD4+ and CD8+ FOXP3+ T cells instead of IFN-y produced by T cells^[40]^. Duffy et al. concluded that MSCs consistently suppress harmful autoimmune Th1 cells responses by predominantly indirect mechanisms, including modulation of antigen-presenting DCs and promotion of naturally occurring or induced FOXP3-expressing Treg^[24]^.

### T helper type 2 cells (Th2)

In the presence of IL-4 in addition to IL-5, IL-9, IL-10 and IL-13, CD4+ T cells differentiate into Th2 cells^[15,32]^. Th2 play a key role in the host defense against extracellular parasites, in recruiting eosinophils and by switching immunoglobulins into IgG1 and IgE in B cells^[15,41]^. Examples of dysregulated Th2 cell responses are associated with allergic diseases such as asthma^[42]^ and rhinitis^[43–45]^. In the presence of allogeneic MSC, a decreased number of infiltrating eosinophils, suppression of IgE induction, reduction of IL-4 and IL-13 production and increases in IL-10 and CD4+ FOXP3 T cells expression have been reported in a mouse model of airway inflammation^[44]^.

### T helper type 17 cells (Th17)

Th17 cell differentiation occurs in the presence of IL-17A in addition to IL17F, IL21 and IL22. Th17 is a pro-inflammatory phenotype that provides protection against fungi and Gram-negative bacteria via neutrophils recruitment^[15,46,47]^. Dysfunctions in Th17 have been associated with multiple inflammatory disorders, such as rheumatoid arthritis, multiple sclerosis and Crohn disease^[13]^. The Interaction of Th17 with MSCs in an inflammatory milieucauses downregulation of Th17 cell-specific factors and upregulation of FOXP3 Treg and IL-10 producing cells^[24,28]^. Furthermore, X Han et al. has recently presented a novel immunosuppressive concept of IL-17 in the presence of MSCs. They suggest that IL-17 enhanced the *in vivo* immunosuppressive effect of MSCs on T cell proliferation in an iNOS-dependent manner^[49]^.

### Cytotoxic T lymphocytes (CTLs)

CD8+ CTLs are a type of T cell that is primarily activated by antigen-dendritic cells such as dendritic cells (DCs). Once activated, CTLs are capable of inducing cell death through cell-to-cell encounter and by the secretion of cytotoxic granules. These functions allow CTLs to play a potent role against virus-infected cells and tumor cells. When in contact with MSCs during the primary stimulation phase, MSCs inhibit the CTL-associated cell lysis. Whereas, if CTLs are in contact at the cytotoxic effector phase, MSCs are unable to suppress the cell lysis^[29,50–52]^. As highlighted in a review by Duffy et al.^[24]^, MSCs present both direct and indirect suppressive effects on the generation of antigen-specific CTLs and may enhance the emergence of CD8+ Treg’s but do not significantly inhibit the immune surveillance functions of preexisting CD8+ memory T cells.

### Regulatory T cells (Treg)

Treg is a subtype of CD4+ T cell, characterized by expression of IL-2 receptors on the surface and intracellular transcription factor FOXP3. Treg play an important immunosuppressive role in the activation, differentiation and effector function of the other Th cell subtypes through the release of soluble factors and by cell-cell contact^[15]^. As described before, the interaction with MSCs, increases the number and activity of Treg and the expression of IL-10 while suppress Th1, Th2 and Th17^[53–55]^.

### MSC and Dendritic Cells

Dendritic Cells (DC) are the most important antigen presenting cells specialized in the uptake, transport, and presentation of antigens and have the unique capacity to stimulate naive and memory T cell in the body^[56]^.These cells are derived from bone marrow CD34+ cells *in vivo* or can be grown *in vitro* from monocytes stimulated with granulocyte macrophage colony-stimulating factor (GM-CSF) and IL-4^[57]^, or from CD34+ hematopoietic progenitors in presence of GM-CSF and TNFa^[58]^. DC play a key role in the initiation of primary immune response and in tolerance depending on the activation and maturation stage of DC^[59]^. Their main function is to process and present antigens to virgin and memory T cells, B lymphocytes and NK cells. Exposure to locally produced cytokines or microbial components, promote the maturation, characterized by upregulation of MHC-II, co-stimulatory molecules CD80 and CD86, migration to lymphoid tissue and production of IL 12. Otherwise, tolerance is observed when antigens are presented by immature or semi-mature DC^[60,61]^.

MSCs can affect the recruitment, maturation, function and homing of DC^[62]^. Nauta et al. on 2006 showed that hMSC were able to significantly reduce monocyte differentiation into DCs, by decreasing upregulation of CD80 and CD86, CD1a, CD83 CD40, and HLA DR^[60]^. This inhibition is performed reversibly via either intercellular contact or soluble factors (Figure 1) as monocytes differentiate normally at the removal of MSCs. However, MSC do not affect direct LPS-induced maturation of DCs in co-cultures^[63,64]^. Furthermore, mature DC co-culture with MSCs show reduced expression of HLA-DR, CD1a, CD83, CD80 and CD86 and down-regulation of IL-12 suggesting their skew towards an immature status. The decreased production of IL-12 is associated with tolerance and anergy of T cells^[65]^. MSCs also cause the LPS-induced mature DC to increase the production of interleukin 10 (IL-10) and to decrease the tumor necrosis factor a (TNFa) secretion, inducing a more anti-inflammatory or tolerant phenotype^[65]^.

### MSC and natural killer (NK) cells

NK cells are a type of lymphocyte critical to the innate immunity response. These cells play a key role against viral infections and tumors^[66,67]^. NK cells perform their effector function through the secretion of cytokines, such as IFNy, TNFa, IL-10, and GM-CSF, and possess cytokine activity both spontaneous and antibody-dependent. NK cells play a pivotal role in the equilibrium of signals transmitted by activator and inhibitor receptors that interact with specific HLA molecules on target cells^[68]^. The outcomes of the interaction between MSCs and NK cells depend on the state of NK cell activation and/or cytokines present in the milieu. Thus it might result in altered cell function and/or survival in either one or the other cell type^[63,69,70]^.

MSCs have been shown to not only affect the phenotype and proliferation of IL-15-induced NK cell without inducing cell death, but also the cytotoxic potential and cytotoxicity against HLA-class I negative expressing targets and/or HLA-class I mismatched of NK cells^[63,71]^. MSCs can inhibit the IL-2 induced proliferation of resting, inactivated NK cells, through the synergistic activity of the soluble factors IDO and PGE2^[63,72]^ and other factors such as TGFb, IL-10 and HGF may play additional roles^[73]^.

MSCs also had an inhibitory effect on proliferating activated NK cells, through the activity of Serine Protease Inhibitor B9 (SERPINB-9), which is a major defense against granzyme B-mediated lysis and of MCP-1, that inhibits perforin expression^[74]^. On the other hand, NK cells can efficiently kill both allogeneic and autologous MSCs. The surface expression of low levels of HLA class I molecules and the expression of activating NK receptor ligand, such as Poliovirus Receptor and MHC class I polypeptide-related sequence A, favors the induced NK-mediated lysis of MSCs^[63,75]^.

### MSC and B Lymphocytes

B lymphocytes are white blood cells involved in humoral immunity components of the adaptive immune system. These cells are specialized for antibody production. Only a limited number of studies have been published regarding the modulatory effects of MSCs on B lymphocytes in humans^[76]^. However, MSCs inhibit the proliferation by arrest of cell cycle G0/G1 without inducing apoptosis and their differentiation into plasma cells and subsequent Ig formation as IgG, IgA, IgM are diminished^[77,78]^. Rosado et al demonstrated that cell-to-cell contact between CD3+ T cells and MSC’s is crucial to inhibit B-cell proliferation and antibody secretion^[79]^. MSCs also inhibit the homing molecules CXCR4, CXCR5, CCR7 modifying the chemotactic properties of B-cells^[77]^.

## MSC and the Paracrine Role of Soluble Factors

Several soluble factors produced by MSCs have been described as having direct influence in being able to suppress the classical proinflammatory markers and shift the immune system toward an anti-inflammatory phenotype. Below we describe the immunomodulation effects of TGF, HGF, IDO, PGE2, IL-6, IL-10, NO, HLA-G5, LIF, Gal-1, Gal-3 and Gal-9 in an inflammatory environment. (Figure 1)

### Transforming growth factor (TGF)-β1 and hepatocyte growth factor (HGF)

Transforming growth factor(TGF) - β1 and hepatocyte growth factor (HGF) are constitutively and synergistically expressed by MSC^[80]^. They play an important rolein immunomodulation of alloantigen-activated T-lymphocytes. It has been shown by Di Nicola et al that neutralizing antibodies to HGF and TGF-β restored the proliferative response^[81]^. HGF also induces MSC mobilization and recruitment to damaged tissuesin addition to a mitogenic and anti-apoptotic activity in various epithelial cells and promotes hematopoiesis^[82,83]^. TGF-β is specifically involved in the generation of CD4+ CD25+ Foxp3+ Treg and in the decreased proliferation of NK cells^[19]^. Whereas HGF markedly suppressed IFN-γ and TNF-α expression and decreased the serum IL-12^[83]^.

### Indoleamine-2,3-dioxygenase(IDO)

Although not constitutively expressed by MSC’s, Indoleamine-2,3-dioxygenase (IDO) can be induced by IFN-γ^[84]^. IDO is an enzyme that catabolizes L-tryptophan along the kynurenine pathway^[85]^, thereby depleting an essential amino acid from the local environment. Thus tryptophan depletion or, a build-up of kynurenine, inhibits allogeneic T cell responses to major histocompatibility complex (MHC)-mismatched allografts^[86]^ and to autoantigens in animal models of disease^[87,88]^. IDO also participates in the inhibition of maturation and functional activity of DCs, in the decrease of proliferation and cytotoxic activity of IL-2-mediated NK cells in the inhibition of Th17 differentiation, and in the generation of Foxp3+ Tregs^[28,89,90]^.

### Prostaglandin E2 (PGE2)

PGE2 is a fundamental homeostatic factor derived from arachidonic acid, synthesized by cyclooxygenases COX1, COX2 and prostaglandin synthetase^[91]^. Several studies have shown the ability of PGE2 to promote the induction of suppressive IL-10, IL-6 and IL-4; to directly suppress the differentiation of monocytes into DCs, to stimulate the proliferation and cytotoxic activity of IL-2 mediated NK cells and to promote the differentiation of Tregs^[92–95]^. PGE2 also prevents the differentiation of naive T cells into pro-inflammatory Th17. Thus, PGE2 is an essential homeostatic factor that plays key role in MSC-mediated immunomodulation.

### Interleukin-6 (IL-6)

Interleukin-6 (IL-6) amplifies the immunosuppressive effects of MSCs and may induce COX2 function in the generation of PGE2 and iNOS activity, enhancing the production of nitric oxide (NO)^[96,97]^. Thereby, IL-6 dependent NO, and IL-6-dependent PGE2, may act systemically suppressing the host immune response through a shift in the Th1/Th2 cell balance and locally by inhibiting generation and maturation of dendritic cells and enhancing the generation of Treg cells^[60,98,99]^.

### Interleukin-10 (IL-10)

Interleukin-10 (IL-10)expression by MSCs remains controversial^[100,101]^; IL – 10 has a known immunomodulatory role in T cells where it promotes the shift Th1/Th2 balance towards the Th2 phenotype and contributes to the proliferation of Treg^[99]^. IL-10 downregulates Th1 cytokine expression (and stimulated the expression of HLA-G5, which is another important soluble factor expressed by MSCs that it is described below^[102]^. It can also antagonize IL-12 during the induction of an inflammatory response, thus decreasing the maturation and function of DCs^[103]^.

### Nitric oxide (NO)

Nitric oxide (NO) is a bioactive molecule produced by NO synthases (NOSs), of which there are 3 subtypes: inducible NOS (iNOS), endothelial NOS (eNOS), and neuronal NOS (nNOS)^[104]^. iNOS expression is inducible and plays a major role in immune regulation^[105]^. Sato et al found in a mouse model that Stat5 phosphorylation in T cells is suppressed in the presence of MSCs and that NO is the key component involved in the suppression^[106]^.

### Human leukocyte antigen-G molecules (HLA-G5)

Human leukocyte antigen-G molecules (HLA-G5) are a soluble isoform of the nonclassic HLA-G molecules, which are MHC-like protein characterized by their low polymorphism expression pattern. HLA-G5 is secreted by MSCs and is IL-10 dependent, direct contact between MSC and T cell is required to obtain a positive feedback loop and thereby a full HLA-G5 secretion^[102,107]^. This soluble molecule has been directly linked to the tolerogenic ability of MSCs to induce the expansion of CD4+ CD25 high FOXP3+ Treg cells^[108]^. HLA-G5 has also been shown suppress T cell proliferation and decrease the cell-mediated cytotoxicity and IFNy secretion by NK^[102]^.

### Leukemia inhibitory factor (LIF)

Leukemia inhibitory factor (LIF) is a functional glycoprotein cytokine that participates in both the humoral and cellular immune response^[109]^. Furthermore, LIF plays an essential role in establishing pregnancy by enabling an allogeneic fetus to avoid rejection by the mother^[110]^. LIF also has a role in the regulation of transplantation tolerance *in vivo*^[111]^. LIF is constitutively secreted by hMSCs. Nasef et al have shown that the use of LIF-neutralizing antibodies decrease Foxp3+ Treg cells, thereby suggesting the involvement of LIF in the generation of Treg cell. They also have found a positive correlation between LIF and HLA-G gene expression by MSCs^[109–112]^.

### Galectins (Gal)

Galectins (Gal) are a family of soluble lectins expressed in various tissues characterized by their high binding affinity to b-galactoside residues. The degree of conservation of their structure sequence across different species characterizes their involvement in the regulation of cellular homeostasis, including many roles in innate and adaptive immunity^[113]^, Among the 15 known subtypes, several galectins have been implicated in MSC-mediated immune regulation described below^[114]^.

### Gal-1

Galectin-1 is a highly expressed intracellular protein in MSCs with diverse functions. It has antiproliferative effects on activated T cells^[115]^ and also supports the survival of naïve T cells^[116]^. Gieseke et al. demonstrated the key immune modulator role that galectin-1 plays on different effects on lymphocyte sub populations and their cytokine profile. This was done with the use of specific knockdown experiments in human MSCs^[117]^. Galectin-1 was found to inhibit the secretion of cytokines typical of Th1 and Th17 cells while promoting Th2-type cytokine secretion^[116]^. Furthermore, galectin-1 was shown to modulate the release of TNFa, IFNy, IL-2 and IL-10 in graft versus host disease^[117,118]^. Importantly galectin-1 promotes the generation of tolerogeneic DC^[119]^. In addition, during feto-maternal tolerance, galectin-1 prevents fetal loss in stress-challenged pregnancies by modulating the Th1/Th2 cytokine balance and by inducing tolerogenic cells^[120]^.

### Gal-3

Over the past decade, galectin-3 has been shown to be an integral component in the immunosuppressive capacity of MSCs. Galectin-3 has the ability to impair the function of DC, which can in turn inhibit T cell function^[121]^. It also induces both phosphatidylserine (PS) exposure and apoptosis in primary activated human T cell^[22]^.

### Gal-9

Galectin-9 is a 36 kDa tandem-repeat galectin that is upregulated by MSCs in an inflammatory environment. This subtype has been shown to maximize the immunomodulatory potential of MSCs, by inhibiting the proliferation of T cells and B cells. Galectin-9 contributes to the suppression of antigen triggered immunoglobulin release^[123]^.

The abundance of mediators identified suggests that MSCs develop different immunosuppressive mechanism under different disease conditions. Overall, it is now well established that MSCs exert potent and diverse modulatory effects on the immune system, most of which are suppressive in nature, and of potential therapeutic value.

## Animals Studies with MSCs

Several studies have documented the dramatic clinical improvements, observed in animal models, and by using systemically introduced MSCs as a therapy of organ injury and immune modulation(Table 1). MSCs can be safely administered in animals and contribute to improved organ function following (lung fibrosis animal study) and account for the beneficial immunomodulatory effects from MSCs. Some examples are described below and summarized in Table 1.

Graft-versus-host disease (GvHD) is a life threatening complication following allogeneic transplantation of hematopoietic stem cells in many malignant and non-malignant disorders. Characterized by dysregulation of inflammatory cytokines and activated donor cells which attack recipient organs and tissues. The first-line treatment is currently steroids. However, in patients with acute or severe, steroid-resistant GvHD the outcomes are poor. In recent years, MSCs have been successfully applied to mouse models of GvHD. These studies have shown increased in the survival rate from the mice, decreased immune cell infiltration and upregulation of anti-inflammatory cytokines^[105,125]^. Systemic Lupus Erythematosus (SLE) is an autoimmune inflammatory disease with multi-organ involvement. Due to the observations presented by Sun et al. from MSCs derived from SLE mouse, the use of allogeneic rather than autologous MSCs are suggested for SLE^[135,136]^. The transplantation of umbilical cord MSCs was found to decrease Th1 cytokines (IFN-y, IL-2) and pro-inflammatory cytokines (TNF-a, IL-6, IL12) and increase Th2 cytokines (IL-4, IL-10). In murine models, MSC therapy ameliorates disease activity, improves serologic markers and certain clinical symptoms such as renal function. Thus, supports the possibility of using umbilical cord MSCs in the treatment for SLE^[127]^.

An experimental autoimmune encephalomyelitis has been used as a model for multiple sclerosis (MS). MS is a chronic inflammatory de-myelinating disease of the central nervous system. A potential therapy to enhance the clinical manifestations has been shown through the intravenous administration of MSCs. When MSCs were used at the disease onset, or at the peak, beneficial effects were exhibited. Whereas no improvement was observed when MSC therapy was used during the chronic phase. MSCs decreased the immune cell infiltration and demyelination in the central nerve system by inducing tolerance to myelin oligodendrocyte glycoprotein in addition to the decreased in the CD4+ T cell and IL-17 cytokine^[128,129]^. Thus, supporting the immunomodulatory role of MSC for the therapy in MS.

A remarkable property of MSC in the treatment of autoimmune type I diabetes significantly delayed the onset of diabetes in non-obese diabetic mice. Injections of MSC into mice were capable to protect islet mass fusion, as supported by insulin staining, islet morphology, and lymphocyte infiltration. Promising results were shown by Fiorina et al in temporarily reversing the hyperglycemia for more than 2 months, in new-onset diabetic mice^[130]^.

In addition to autoimmune diseases, MSC are also capable of inhibiting the inflammation and the apoptosis in the bleomycin-induced pulmonary fibrosis model by decreasing the accumulation of connective tissue^[131,132]^. This suggests that cell-based therapies may be potential therapeutic approaches for lung regeneration and normal wound healing after injury. In mouse model of allergic inflammation^[43]^ and asthma^[133]^, MSCs were shown to provide a beneficial effect by decreasing the eosinophilic Th2 inflammatory response, evidenced histologically and by IgE serum concentrations^[134,135]^.

## Clinical Applications of MSCs

Understanding of the underlying biological mechanisms of MSCs in modulating the immune response and tissue regeneration in preclinical studies triggered an explosive interest from numerous research groups to explore its role in clinical settings. More than 600 clinical studies have been registered on the clinical trial database (www.clinicaltrials.gov) in the hope of dissecting the therapeutic roles from MSC in various human diseases.

Beneficial effects of MSCs had been found in the treatment for many immune-associated diseases. The immunomodulatory effects of MSCs on clinical trials are summarized in Table 2. For instance, MSC have successfully reduced the incidence and severity in patients diagnosed with GVHD with severe steroid resistance^[136–139]^. Similarly, in autoimmune diseases such as SLE and MS, the application of MSCs was safe and effective. No toxic effects were observed. The immediate immunosuppressive capacity from MSCs has been described^[140–145]^. However, the specific mechanism through which improved the patient condition is unknown.

Recent studies have shown that MSCs significantly reduced the insulin requirement in patients with diabetes type I after the administration of Wharton Jelly derived (Umbilical cord)-MSC’s^[146]^. Similar results were shown in an open-labeled clinical trial after co-transplantation of adipose derived-MSC and hematopoietic stem cells in the same population^[147,148]^. In the treatment of diabetes type 2, the administration of placenta-derived MSCs improved the renal and cardiac function and the daily mean dose of insulin was reduced in 10 patients^[149]^. Another study using Wharton Jelly derived MSC also demonstrated improvements in the metabolic control and beta cell function^[150]^. Although there are multiple preclinical studies suggesting that MSC may be efficacious in the treatment of idiopathic pulmonary fibrosis (IPF), very few clinical investigations have been reported. Chambers DC et al and Tzouvelekis et al. showed that intravenous MSC therapy has satisfactory short-term safety profile in moderately severe IPF and improvements in quality of life parameters. However, no improvements in lung function indicators were shown^[151,152]^.

## Challenges

MSCs exhibit great potential in most preclinical and clinical data. However, many questions remain to be solved. The optimal source of MSCs, the optimal time window, the dosage, the route and frequency of MSC administration, the post-transplantation safety and the long-term prognosis have still not been determined^[153]^. Although the use of autologous, allogeneic and xenogeneic MSCs have shown great response and a great promise for novel therapeutic approaches, it is important to pursue comparative studies to determine whether MSCs from alternative sources operate with the identical immune regulatory mechanisms as BM-MSCs, which are used in cellular therapy. These studies will be vital in determining alternative sources of MSCs for their potential implementation at the clinical level.

Furthermore, the lack of standardized culture configuration together with the heterogeneous populations produced makes it difficult to compare the results from different studies. In addition, other issues still need to be addressed, such as the amount of immune cell subsets necessary to provide immunomodulation through MSC? Which pathway(s) is/are involved? What is the diverse systemic response towards MSC in different disease settings? Are there any safety issues during further clinical trials? The MSC can be safely and routinely applied in the future only after these unexplored territories have been clarified^[154]^.

Thus, in order to overcome these challenges, standardized protocols for cell culture, differentiation, expansion and cryopreservation, as well as robust quality control systems, need to be in place. These factors in combination with safely preconditioned and genetically modified MSCs may pave the way for the development of an effective and safe cellular therapy for countless human immune disorders.

## Conclusion Remarks

Novel concepts of the immunomodulatory properties of MSCs were described in this review. The mechanisms, by which the MSCs interact within the immune system through the cell to cell interaction, and by releasing soluble factors, are also highlighted. Our current knowledge makes MSCs an important regulator of the immune tolerance and attractive therapeutic target for limiting autoimmune inflammation, preventing allograft rejection and potentiating antitumor responses. Although the results are very promising, an optimal and standardized manipulation, a better understanding of the immune-biology, and the interactions within the microenvironment, needs to be strengthened in order to use them in a universal and effective way in the clinical setting.

## Figures and Tables

**Table 1:** Immunomodulatory effects of mesenchymal stem cell-based therapy on animal studies.

Disease	MSC Source	Species	Route of Administration	Mechanism of MSC effect	Reference
**GvHD**	hUC-MSCs	DBA/2 (H-2Kd) mice	Intravenous	Expression of IDO and TGF-B	Guo J et al, 2011^[124]^
**GvHD**	C57BL/6 mice, NOS^−/−^ or IFNγR1^−/−^ BM-MSCs	C57BL/6, C3H/HeJCr, and F1	Intravenous	Upregulation of inducible nitric oxide synthase (iNOS) and leukocytes chemokine (CXCL9, CXCL10 and CXCL11)	Ren G et al, 2008^[155]^
**Systemic lupus erythematosus**	C3H/Hej mice BM-MSCs	MRL/lpr mice	Intravenous	Downregulation of Th17 levels and increase of Foxp3+cells	Sun L et al, 2009^[125]^
**Systemic lupus erythematosus**	hUC-MSCs	NZB/W F1 mice	Intravenous	Induce the polarization of Th2 cytokine and proinflammatory inhibition.	Chang JW et al, 2011^[127]^
**Autoimmune encephalomyelitis (model of multiple sclerosis)**	Mice BM-MSCs	C57BL/6	Intravenous	Inhibition of T-cell receptor dependent and independent polyclonal stimuli	Zappia E et al. 2005^[128]^
**Autoimmune type 1 diabetes**	Murine BM-MSCs	NOD mice	Intravenous	Inhibition of autoreactive Tcells and increase in the percentage of Tregs and Th2 cytokines	Fiorina P et al, 2009^[130]^
**Asthma**	Balb/c mice BM-MSCs	C57BL/6J mice	intravenous	Increase TGF-beta production	Nemeth K et al, 2010^[133]^
**Allergic rhinitis**	Balb/c mice adipose tissue MSCs	Balb/c mice	Intravenous	Inhibition of eosinophil inflammation via shifting from Th2 to Th1 immune response.	Cho Ks et al, 2010^[135]^
**Pulmonary fibrosis**	Mouse BM-MSCs	Bleomycin mouse model	intravenous	Induce mobilization of endogenous stem cells through GM-CSF and G-CSF.	Rojas et al, 2005^[132]^
**Pulmonary fibrosis**	Mouse BM-MSCs	Bleomycin mouse model	Intravenous	None specific	Ortiz et al, 2003^[131]^

**Abbreviations:** GvHD: graft versus host disease; SLE: systemic lupus erythematosus; MSC: mesenchymal stem cells; hMSC: human mesenchymal stem cells; hUC-MSC: human umbilical cord derived mesenchymal stem cell, BM-MSC: bone marrow derived mesenchymal stem cell.

**Table 2:** Clinical trials using mesenchymal stem cell-based therapy.

Disease	Sample size	Study Period	MSC source	Dosage	Administration Route	Effects	Clinical Trial Stage	Reference
GvHD	55 Adults	60 months	Allogeneic BM-MSCs	0.4–9 × 10^6^/ kg, 1–5 doses	I.V	CR (30/55) PR (9/55), NR (16/55) Increase overall survival in CR, no adverse events	Phase II	Le Blanc K et al, 2008^[137]^
GvHD	31 Adults	28 days	Allogeneic BM-MSCs	2 or 8 × 10^6^/ kg, 1 dose	I.V	CR (24/31), PR (5/31)NR (2/31), no adverse events.	Phase II	Kebriaei P et al, 2009^[136]^
GvHD	2 Children	18 months	UC-MSCs	3.3 – 8.0 × 10^6^/kg, 4 doses	I.V	CR (2), no adverse events.	Case Report	Wu KH et al, 2011^[139]^
GvHD	13 Adults	257 days	Allogeneic BM-MSC	0.9–1.1 × 10^6^/ kg, 2 doses	I.V	CR (2/13) PR (5/13), NR (6/13), no adverse events	Case series	Von Bonin M et al, 2009^[138]^
SLE	40 Adults	12 months	UC-MSCs	1×10^6^/kg, 2 dose	I.V	CR (13/40), P (11/40), NR (16/40), 7 recurrence, no adverse events.		Wang D et al, 2014^[140]^
SLE	35 Adults	21 months	8 receive BM-MSCs and 27 UC-MSCs	1×10^6^/kg/1–3 doses	I.V	CR (33/35), recurrence (2/35), Increase in Treg and decrease of Th17. No adverse events		Li X et al, 2013^[145]^
SLE	87 Adults	27 months (mean)	BM-MSCs and UC-MSCs	1×10^6^/kg/ 1 dose	I.V	CR (43/87)at 4 years, P/NR (44/87), relapse 20/87at 4 years, no adverse events	Phase I/II	Wang D et al, 2013^[141]^
Multiple sclerosis and Amyotrophic lateral sclerosis	MS: 15 Adults ALS: 19 Adults	6 months	Autologous BM-MSCs	MS: 6.32 × 10^7^/kg ALS: 1.74 × 10^7^. 1 dose	Intrathecal and I.V	CR (20/34), P/NR (14/30), no adverse events	Phase I/II	Karussis D et al, 2010^[143]^
Multiple sclerosis	10 Adults	10 months	Autogenous BM-MSCs	(1.1 – 2.0) × 10^6^/kg. 1 dose	I.V	CR (10/10), improvement in visual acuity, visual evoked response latency and optic nerve area. No significant adverse events.	Phase IIA	Connick P et al, 2012^[144]^
Diabetes	41 Adults	24 months	Autologous BM-MSCs	Non state	I.M	Improved painless walking time, ankle-brachial index, transcutaneous oxygen pressure and magnetic resonance angiography. No serious adverse events	Phase I	Lu D et al, 2011^[148]^
Type 1 Diabetes	29 Adolescents	21 months	Allogeneic UC- MSCs	(1.5 – 3.2 × 10^7^/kg	I.V	CR (3/15), P (9/15), NR (3/15) Improved recovery and regeneration of islet B-cells. No serious adverse events		Hu J. et al, 2013^[146]^
Type 1 Diabetes	11	23 months	Allogeneic ADMSCs	4.6 × 10^7^ −2.48 × 10^8^ cells/dose (range)	I.V(Intraportal)	CR (11/11), Gradual decrease in insulin requirements and in Hb1Ac. No adverse events		Vanikar AV et al, 2010^[147]^
Type 2 Diabetes	10 Adults	3 Months	Allogeneic placenta derived MSCs	1.35 × 10^6^/kg, 3 doses	I.V	CR (10/10) Decrease in insulin requirements. No adverse events	Phase I	Jiang R et al, 2011^[149]^
Type 2 Diabetes	22 Adults	12 months	UC-MSCs	1 × 10^6^/kg, 2 doses	I.V	CR (17/22) PR/NR (5/22) Improvement in B cell function, systemic inflammation (IL-6 and IL-1B) and T cells counts (CD3+ and CD4+). Adverse events (2/22)	Phase I/II	Liu X et al, 2014^[150]^
Idiopathic Pulmonary Fibrosis	8 Adults	6 months	Allogeneic Placenta derived MSCs	1 × 10^6^/kg or 2 × 10^6^/kg	I.V	PR/NR (8/8). Small bowel obstruction, left lower lobe consolidation and mild episodes of bronchitis were reported as side effects (3/8)	Phase 1b	Chambers DC et al, 2013^[151]^
Idiopathic Pulmonary Fibrosis	14 Adults	12 months	Autologous AD-MSCs	1.5 × 10^6^/kg, 3 doses	Endobronchial	NR (14/14) Worsening cough and dyspnea were reported as adverse events (2/14)	Phase 1b	Tzouvelekis A et al, 2013^[156]^

**Abbreviations:** GvHD: graft versus host disease; SLE: systemic lupus erythematosus; MSC: mesenchymal stem cells; UC-MSC: umbilical cord derived mesenchymal stem cell, BM-MSC: bone marrow derived mesenchymal stem cell; AD-MSC: adipose derived mesenchymal stem cell; I.V: intravenous; I.M: intramuscular; CR: complete response; PR: partial response; NR: no response.

## Abbreviations

BM: bone marrow
iNOS: inducible nitric oxide synthase
COX2: cyclooxygenase 2
MSC: mesenchymal stem cell
NK: natural killer cell
GvHD: graft versus host diseases
DBT: diabetes
SLE: systemic lupus erythematosus
MS: multiple sclerosis
IPF: idiopathic pulmonary fibrosis
NO: nitric oxide
HGF: hepatocyte growth factor
PGE2: prostaglandin E2
HLA: human leukocyte antigen
IDO: indoleamine 2, 3- dioxygenase
TGF-β: transforming growth factor-β
IFN-γ: interferon-γ
TNF-α: tumor necrosis factor-α
IL-10: interleukin-10
Th1: T helper type 1 cells
Th2: T helper type 2
Th17: T helper type 17
Treg: regulatory T cell
DCs: dendritic cells
CTLA-4: cytotoxic T lymphocyte antigen 4
DC: dendritic cell
PBMC: peripheral blood mononuclear cells
MHC: major histocompatibility complex
LPS: lipopolysaccharide
GM-CSF: granulocyte macrophage colony-stimulating factor
LIF: Leukemia inhibitory factor
